# Reduced GABA concentration in patients with white matter hyperintensities

**DOI:** 10.3389/fnins.2023.1320247

**Published:** 2023-12-14

**Authors:** Xin Wang, Caihong Wang, Peifang Miao, Ying Wei, Liangjie Lin, Zhen Li, Yong Zhang, Jingliang Cheng, Cuiping Ren

**Affiliations:** ^1^Key Laboratory for Functional Magnetic Resonance Imaging and Molecular Imaging of Henan Province, Department of MRI, The First Affiliated Hospital of Zhengzhou University, Zhengzhou, China; ^2^Clinical and Technical Support, Philips Healthcare, Beijing, China; ^3^Department of Interventional Radiology, The First Affiliated Hospital of Zhengzhou University, Zhengzhou, China

**Keywords:** white matter hyperintensities, spectral editing, ^1^H-MRS, GABA, MEGA-PRESS

## Abstract

To investigate potential alterations of white matter hyperintensities (WMHs) on *J*-edited MR spectroscopy (MRS) measures of the primary inhibitory neurotransmitter γ-aminobutyric acid (GABA). Twenty-four WMHs patients and 20 healthy controls (HCs) were recruited to undergo magnetic resonance spectroscopy (MRS) scan at 3T from voxels in left centrum semiovale white matter, using the MEGA point resolved spectroscopy (MEGA-PRESS) technique with the MATLAB-based Gannet tool to estimate GABA+ co-edited macromolecule (GABA+) levels and using Tarquin software to estimate levels of glutamate + glutamine (Glx), total N-acetylaspartate (tNAA), total choline (tCho), and total creatine (tCr). Independent *t*-tests or Mann-Whitney *U*-tests were used to test group differences between WMHs and HCs. Additionally, WMHs patients were divided into mild and moderate-severe WMHs subgroup according to the Fazekas scale. Analysis of variance (ANOVA) and *post-hoc* tests were used among WMHs subgroups and HCs. We found there was a significant reduction in GABA+ levels (*p* = 0.018) in WMHs patients compared with healthy controls. In subgroup analyses, there was also a significant reduction of GABA+ levels in moderate-severe WMHs subgroup (*p* = 0.037) and mild WMHs subgroup (*p* = 0.047) when compared to HCs. Besides, the moderate-severe WMHs subgroup had significantly higher levels of tCho compared with healthy controls (*p* = 0.019). In conclusion, reduced GABA+ levels in WMHs patients and elevated tCho levels in moderate-severe WMHs were observed when compared with HCs. These results demonstrate that abnormalities of the GABAergic system and choline metabolism may contribute to the pathogenesis of WMHs.

## Introduction

White matter hyperintensities (WMHs), was called Leukoaraiosi (LA) initially (Alexander, 1987). It presents as a symmetrical, diffuse speckled or patchy irregular hypodense area in the paraventricular and centrum semiovale white matter on CT images and hyperintensity on T2-weighted (T2WI) or/and fluid-attenuated inversion recovery (FLAIR) MRI. WMHs was classified into two subtypes based on possible different pathogenic mechanisms: periventricular white matter hyperintensities (PVWMHs) and subcortical white matter hyperintensities (SCWMHs) (Kim et al., 2008). WMHs was one of the earliest and most established markers of cerebral small vessel disease (SVD), with a pathophysiological substrate in demyelination and small artery atherosclerosis. And a recent study shows that increased WHM volume is associated with amyloid accumulation (Shirzadi et al., 2023). WMHs frequently raise the risk of stroke, dementia, and mortality in the elderly although they are considered healthy people clinically (Debette and Markus, 2010; Debette et al., 2019), jeopardizing their health and quality of daily life to a certain extent. Investigation of the WMHs is necessary to clarify the progression of SVD and may provide new insights into the mechanism of demyelination produced by ischemic.

Proton magnetic resonance spectroscopy (^1^H-MRS) is a non-invasive imaging technique that quantitatively detects metabolite levels *in vivo*. Conventional MRS at 3T can quantify various brain metabolites including N-acetylaspartate (NAA), choline (Cho), creatine (Cr), and glutamate + glutamine (Glx) etc. Previous studies have detected decreased metabolic ratios of NAA/Cho in patients with SVD (Hund-Georgiadis et al., 2001; Wang et al., 2012), but the ratio does not explicitly elaborate whether it is the numerator or the denominator that has altered. Recent ^1^H-MRS research revealed that absolute quantification outperforms relative ratio in distinguishing patients from healthy controls (Watanabe et al., 2008).

Gamma-aminobutyric acid (GABA) is the main inhibitory neurotransmitter in the human central nervous system. Quantitative measurement of intra-cerebral GABA using conventional ^1^H-MRS is restricted because the low content of GABA (Govindaraju et al., 2000) and the signal of GABA greatly overlap with other main metabolites such as glutamate (Glu), NAA, Cr and macromolecules (MM). While GABA concentration can be reliably measured *in vivo* by *J*-edited MRS (Mescher et al., 1998). Mescher-Garwood point resolved spectroscopy (MEGA-PRESS) is the most common used *J*-edited MRS approach for quantifying GABA in human brain, which made the signal of GABA largely free from overlap with more abundant molecules, and it has been applied successfully to measure GABA in several neurologic and psychiatric disorders (Marenco et al., 2016; Chen et al., 2019) and healthy control subjects (Gao et al., 2013; Frank et al., 2022).

However, to date, there has been a paucity of studies exploring changes in GABA concentration in WMHs patients. The primary purpose of the present study was to determine whether there were abnormalities in the level of GABA, and alterations in the levels of NAA, Cr, Cho, and Glx in WMHs patients.

## Materials and methods

### Subjects

Twenty-four patients with WMHs (WMHs group, 9 males/15 females, mean age 61.04 ± 5.73 years) were recruited for this study. All WMHs patients were defined as paraventricular or subcortical cerebral white matter hyperintensities. Severity assessment of WMHs was evaluated using the Fazekas scale (Fazekas et al., 1987) to assess the scores of PVWMHs and SCWMHs simultaneously for each patient: (1) PVWMHs: 0 = absence, 1 = “caps” or pencil-thin lining, 2 = smooth “halo,” and 3 = irregular periventricular hyperintensity extending into the deep white matter; (2) SCWMHs: 0 = absence, 1 = punctate lesion, 2 = beginning of fusion, and 3 = extensive areas of fusion. Diagnosing WMHs and assessing the degree of severity were completed by two trained investigators in blinded evaluation, with disputes being settled by negotiation. Patients were divided into two subgroups according to the sum of PVWMHs and SCWMHs scores: mild (Fazekas 1–2) and moderate-severe (Fazekas 3–6). Finally, fourteen patients with WMHs met the criteria for the mild subgroup, and ten patients with WMHs met the criteria for the moderate-severe subgroup. At the same time, the occurrence of hypertension, hyperlipidemia, diabetes mellitus and cognitive impairment in the patient group was also recorded.

The exclusion criteria for patients were the following: (1) a history of head wounds, neurologic, or psychiatric disorder; (2) a history of substance abuse; (3) White matter leukoencephalopathy of non-vascular origin immunological demyelinating, metabolic, toxic, other; and (4) MRI conventional contraindications. Additionally, twenty age- and gender-matched healthy controls (HCs group, 7 males/13 females, mean age 58.1 ± 5.98 years) were recruited from local communities.

All participants gave informed consent before starting the study, which was approved by the medical research ethics committees of The First Affiliated Hospital of Zhengzhou University (ChiCTR1900027064).

### Imaging acquisition

All MRI and MRS scans were performed using a 3T scanner (Ingenia CX, Philips Healthcare, Best, Netherlands) with a 32-channel phased-array head coil. The three-dimensional T1-weighted imaging was scanned and used as the MRS voxel localizer with scanning parameters as follows: repetition time (TR) = 8.9 ms; echo time (TE) = 4.1 ms; slice thickness = 0.9 mm; field of view = 256 mm × 243 mm; flip angle = 8°; voxel size = 0.9 mm × 0.9 mm × 0.9 mm. T2-FLAIR images (TR = 4600 ms; TE = 340 ms; slice thickness = 1 mm; field of view = 240 mm × 240 mm; voxel size = 1 mm × 1 mm × 1 mm) were acquired in order to assess the severity of brain lesions.

**FIGURE 1:**
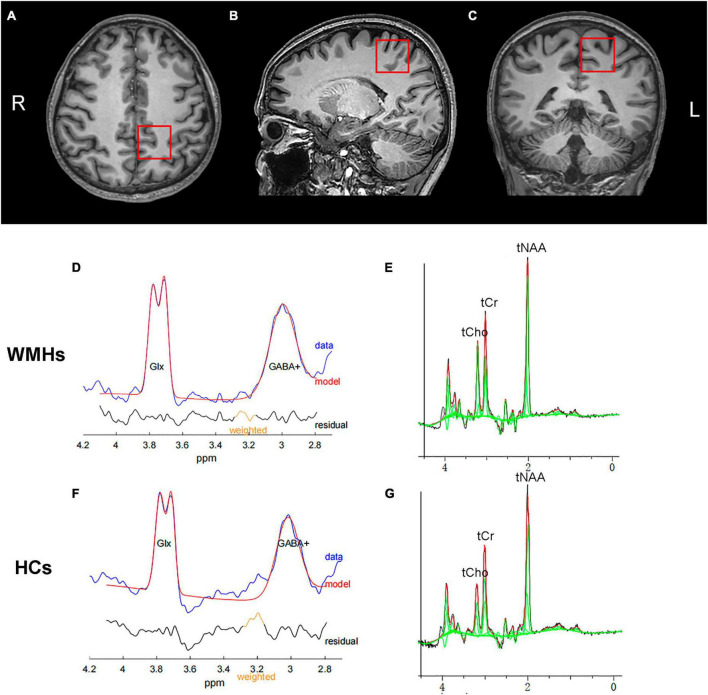
A example of MRS voxel placement and MEGA-PRESS spectrum. T1-weighted images showing single voxel placement centered on left centrum semiovale in the axial **(A)**, sagittal **(B)**, and coronal **(C)** projections. Representative typical GABA-edited spectra from the 1 patient with WMHs **(D)** and 1 healthy control **(F)**, showing the well-resolved edited GABA+ and coedited Glx peaks at 3.0 and 3.8 ppm, respectively. Representative GABA-edited OFF spectra from the WMHs group **(E)** and HCs group **(G)** for quantification of domainal metabolites including tNAA, tCho, and tCr.

Metabolic spectra were obtained from one single voxel at the left centrum semiovale in patients and controls with voxel size of 3 cm × 3 cm × 3 cm as shown in Figure 1. The voxels were positioned in a manner as to avoid the lateral ventricles and skull.

The MEGA-PRESS *J*-editing sequence was used for the 3 ppm resonance of GABA measurement, with parameters as: TR/TE = 2000/68 ms; number of signal averages = 192 (96 for both ON and OFF acquisitions); spectral width = 2000 Hz, and acquisition time = 396 s. During odd-numbered acquisitions, two frequency-selective editing pulses were applied to the GABA-^3^CH2 resonance at 1.9 ppm, affecting the weakly *J*-coupled triplet peak of the GABA-^4^CH2 at 3.0 ppm (EDIT-ON). During even-numbered acquisitions, the same pulses were applied at 7.5 ppm, symmetrically to the other side of the water peak (EDIT-OFF). The GABA-edited spectrum was derived from the difference between the EDIT-ON and EDIT-OFF spectra. The variable-pulse power and optimized relaxation delays (VAPOR) scheme was used for water suppression (Tkác et al., 1999). FASTMAP shimming of the volume of interest was performed automatically before each acquisition. For water-scaled metabolite quantification, measurement (32 averages) of the unsuppressed water signal from the same spectroscopic volume was obtained.

### Spectroscopic post-processing and data analysis

As the measure of signal at 3.0 ppm acquired with the MEGA-PRESS sequence contains contributions from both mobile brain macromolecules (MM) and homocarnosine (Rothman et al., 1997), the detected signal is referred as GABA+ rather than GABA. The GABA+ data were processed using the Gannet 3.0 toolkit in Matlab 2020b (Mathworks) (Edden et al., 2014). Gannet used a single-Gaussian model to fit the edited GABA+ peak at 3.0 ppm, a two-Gaussian model to fit the edited Glx peak at 3.8 ppm, and a Gaussian-Lorentzian model for the water signal at 4.7 ppm. A total of 3 Hz exponential line broadening was applied. GABA+ levels was evaluated relative to water amplitude in institutional units (IU). Gannet also provides normalized residual fitting errors of GABA+ , which is used to assess spectral quality. Only spectra that had fitting errors of GABA+ below 10% were included in the statistical analysis. Each spectroscopic voxel was coregistrated to the 3D T1-weighted brain image, and segmented as gray matter (GM), white matter (WM), and cerebrospinal fluid (CSF) using the “Coregister” and “New Segment” functions of the Statistical Parametric Mapping toolbox (SPM12) within the Gannet toolbox. The concentration of GABA in CSF was assumed to be negligible, and the CSF corrected GABA+ level was finally quantified. The MEGA-PRESS EDIT-OFF sub-spectrum is similar to a PRESS spectrum. The level of total NAA (tNAA), total Cho (tCho), and total Cr (tCr) were calculated from a pre-processed GABA+ sequence using Tarquin software. In addition, metabolite relative ratio GABA+ /tCr, GABA+ /tNAA, Glx/tCr, and Glx/tNAA were also calculated in our study.

### Statistical analysis

Data analyses were conducted using the statistical package SPSS 20.0 software (SPSS, Inc., Chicago, IL, United States). Normality tests based on the Shapiro-Wilk test. Two-sample *t*-tests or chi-square tests were used to explore group differences in age and gender between the WMHs and HCs. Independent *t*-tests or Mann-Whitney *U*-tests were used to test group differences in GABA+ , Glx, tNAA, tCho, and tCr levels between WMHs and HCs. Subgroup analysis was conducted using analysis of variance (ANOVA) and *post-hoc* tests among WMHs subgroups and HCs. The threshold of significance was set as *p* < 0.05. All quantitative measurements were presented as mean ± standard deviation.

## Results

### Demographic characteristics of study subjects

The demographic characteristics of participants are described in Table 1. No significant difference in age (*p* = 0.104) and gender (*p* = 0.864) was found between WMHs and HCs groups. There were six subjects with hypertension, three subjects with hyperlipidemia, two subjects with diabetes mellitus, and none with cognitive dysfunction in WMHs patients. Fisher’s exact test showed significant differences in hypertension (*p* = 0.025), there was no significant differences in hyperlipidemia (*p* = 0.239), and diabetes mellitus (*p* = 0.493) between WMHs patients and healthy controls (Supplementary Table 1).

**TABLE 1:** Demographic data, metabolite quantification, and brain tissue segmentation in MRS voxels for WMHs patients and healthy controls.

	WMHs	HCs	Test statistics	*P*-value
Number	24	20		–
Age (years)	61.04 ± 5.73	58.1 ± 5.98	*t* = 1.662	0.104
Female, number (%)	15 (63)	13 (65)	χ^2^ = 0.29	0.864
tNAA	11.27 ± 1.11	11.52 ± 0.89	*t* = −0.821	0.416
tCho	1.79 ± 0.23	1.70 ± 0.19	*t* = 1.348	0.185
tCr	7.74 ± 0.81	7.61 ± 0.59	*t* = 0.6	0.551
GABA+ level (IU)	2.53 ± 0.29	2.74 ± 0.24	*t* = −2.46	0.018
GABA+ fitting errors (%)	4.05 ± 1.02	4.10 ± 0.72	*z* = −0.578	0.563
Glx level (IU)	9.0 ± 2.22	9.34 ± 0.56	*z* = −0.216	0.829
Glx fitting errors (%)	2.86 ± 0.63	3.17 ± 0.62	*t* = −1.532	0.133
FWHM (Hz)	8.64 ± 0.50	8.65 ± 0.51	*t* = −0.338	0.737
GM/(GM + WM) (%)	41.75 ± 8.02	46.7 ± 3.58	*t* = −2.497	0.017

FWHM, full-width at half-maximum; WM, white matter; GABA+, GABA plus co-edited macromolecules; GM, gray matter; HCs, healthy controls; WMHs, white matter hyperintensities.

### Tissue segmentation and data quality

The mean GM tissue fractions GM/(GM + WM) for the WMHs and HCs groups were 41.75 and 46.7%, respectively, with significant difference (*p* = 0.017) (Table 1).

The mean fitting errors of GABA+ were 4.05 and 4.10%, and the mean fitting errors of Glx were 2.86% and 3.17% in the WMHs and HCs groups, respectively. These were also not significantly different (GABA+ , *p* = 0.563; Glx, *p* = 0.133).

The full-width at half-maximum (FWHM) of all VOIs was below 10 Hz both in WMHs and HCs groups. The mean FWHM were 8.64 and 8.65 Hz for WMHs and HCs groups, respectively, without significant differences (*p* = 0.737) (Table 1).

For subgroup analyses, ANOVA analyses showed significant differences in GM/(GM + WM) among WMHs subgroups and HCs group (*p* = 0.006). The multiple comparisons showed there was no significant difference between mild subgroups and HCs (*p* = 0.138) but there was between moderate-severe WMHs subgroups and HCs (*p* = 0.002) (Tables 2, 3). No significant difference in GM/(GM + WM) between moderate-severe WMHs and mild WMHs subgroups was found (*p* = 0.095) (Table 4). ANOVA analyses showed no significant differences in mean fitting errors of GABA+ (*p* = 0.906), mean fitting errors of Glx (*p* = 0.320), and FWHM (*p* = 0.931), respectively, among WMHs subgroups and HCs group.

**TABLE 2:** Metabolite quantification in mild WMHs subgroup patients and healthy controls.

	Mild WMHs	HCs	Test statistics	*P*-value
Number	14	20		–
Age (years)	59.79 ± 5.32	58.1 ± 5.98	t = 0.845	0.393
Female, number (%)	11 (79)	13 (65)	χ^2^ = 0.731	0.467
tNAA (mM)	11.35 ± 1.03	11.52 ± 0.89	*t* = −0.494	0.624
tCho (mM)	1.73 ± 0.25	1.70 ± 0.19	*t* = 0.296	0.769
tCr (mM)	7.68 ± 0.97	7.61 ± 0.59	*t* = 0.256	0.8
GABA+ level (IU)	2.57 ± 0.22	2.74 ± 0.24	*t* = −2.076	0.047
GABA+ fitting errors (%)	4.11 ± 1.26	4.10 ± 0.72	*z* = −0.666	0.505
Glx level (IU)	8.80 ± 2.75	9.34 ± 0.56	*z* = −0.76	0.447
Glx fitting errors (%)	2.89 ± 0.72	3.17 ± 0.62	*t* = −1.155	0.256
FWHM (Hz)	8.62 ± 0.45	8.65 ± 0.51	*t* = −0.396	0.695
GM/(GM + WM) (%)	44.06 ± 6.35	46.7 ± 3.58	*t* = −1.521	0.138

FWHM, full-width at half-maximum; WM, white matter; GABA +, GABA plus co-edited macromolecules; GM, gray matter; HCs, healthy controls; WMHs, white matter hyperintensities.

**TABLE 3:** Metabolite quantification in moderate-severe WMHs subgroup patients and healthy controls.

	Moderate-severe WMHs	HCs	Test statistics	*P*-value
Number	10	20		–
Age (years)	62.8 ± 6.09	58.1 ± 5.98	*t* = 2.017	0.053
Female, number (%)	4 (40)	13 (65)	χ^2^ = 0.072	0.789
tNAA (mM)	11.14 ± 1.25	11.52 ± 0.89	*t* = −0.954	0.348
tCho (mM)	1.88 ± 0.17	1.70 ± 0.19	*t* = 2.483	0.019
tCr (mM)	7.83 ± 0.55	7.61 ± 0.59	*t* = 0.973	0.339
GABA+ level (IU)	2.49 ± 0.37	2.74 ± 0.24	*t* = −2.201	0.037
GABA+ fitting errors (%)	3.96 ± 0.60	4.10 ± 0.72	*t* = −0.512	0.613
Glx level (IU)	9.29 ± 1.23	9.34 ± 0.56	*z* = −0.552	0.581
Glx fitting errors (%)	2.83 ± 0.51	3.17 ± 0.62	*t* = −1.43	0.164
FWHM (Hz)	8.66 ± 0.35	8.65 ± 0.51	*t* = −0.141	0.889
GM/(GM + WM) (%)	38.51 ± 9.29	46.70 ± 3.58	*t* = −3.43	0.002

FWHM, full-width at half-maximum; WM, white matter; GABA +, GABA plus co-edited macromolecules; GM, gray matter; HCs, healthy controls; WMHs, white matter hyperintensities.

**TABLE 4:** Metabolite quantification in mild WMHs subgroup and moderate-severe WMHs subgroup patients.

	Mild WMHs	Moderate-severe WMHs	Test statistics	*P*-value
Number	14	10		–
Age (years)	59.79 ± 5.32	62.8 ± 6.09	*t* = 1.289	0.211
Female, number (%)	11 (79)	4 (40)	χ^2^ = 0.974	0.324
NAA (mM)	11.35 ± 1.03	11.14 ± 1.25	*t* = −0.454	0.654
Cho (mM)	1.73 ± 0.25	1.88 ± 0.17	*t* = 1.689	0.105
Cr (mM)	7.68 ± 0.97	7.83 ± 0.55	*t* = 0.437	0.666
GABA+ level (IU)	2.57 ± 0.22	2.49 ± 0.37	*t* = −0.634	0.537
GABA+ fitting errors (%)	4.11 ± 1.26	3.96 ± 0.60	*z* = −0.264	0.792
Glx level (IU)	8.80 ± 2.75	9.29 ± 1.23	*z* = −0.644	0.519
Glx fitting errors (%)	2.89 ± 0.72	2.83 ± 0.51	*t* = −0.209	0.836
FWHM (Hz)	8.62 ± 0.45	8.66 ± 0.35	*t* = 0.183	0.857
GM/(GM + WM) (%)	38.51 ± 9.29	44.06 ± 6.35	*t* = −1.742	0.095

FWHM, full-width at half-maximum; WM, white matter; GABA +, GABA plus co-edited macromolecules; GM, gray matter; HCs, healthy controls; WMHs, white matter hyperintensities.

### Comparisons of metabolite levels between WMHs and HCs groups

**FIGURE 2:**
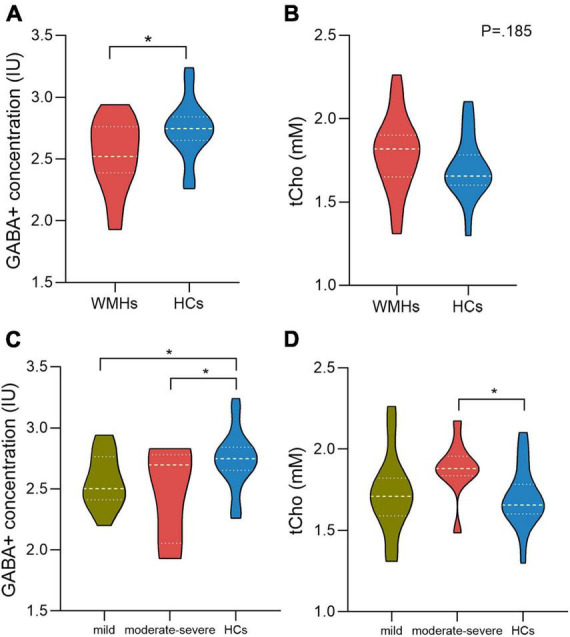
γ-Aminobutyric acid (GABA) + concentration of the WMHs group are significantly decreased compared to HCs **(A)**. There is no difference between WMHs and HCs in the level of tCho **(B)**. For comparison of GABA+ concentration within WMHs subgroups and HCs, mild and moderate-severe WMHs subgroups had significantly lower GABA+ levels compared to HCs while no significant difference between mild WMHs subgroup and moderate-severe WMHs subgroup **(C)**. Moderate-severe WMHs subgroup had significantly increased tCho levels compared to HCs group **(D)**. IU = institutional units. mM= millimolar. *P < 0.05.

The fitting errors of GABA+ and Glx in all spectra were below 10%. Representative metabolite-edited spectra are shown in Figure 1. GABA+ levels (water signal scaled) were significantly lower in the WMHs group compared to the HCs group (*p* = 0.018) (Table 1 and Figure 2A). No significant alteration was observed in levels of Glx, tNAA, tCho, and tCr between the WMHs group and HCs group (Table 1 and Figure 2B). Metabolite relative ratios shows that GABA+ /tCr were significantly lower in the WMHs group compared to the HCs group (*p* = 0.015) (Supplementary Table 2 and Supplementary Figure 1A). There was no significant alteration in GABA+ /tNAA, Glx/tCr, Glx/tNAA (Supplementary Table 2).

### Comparisons of metabolite concentration among WMHs subgroups and HCs group

There were 10 moderate-severe WMHs patients and 14 mild WMHs patients. ANOVA analyses showed significant differences in GABA+ (*p* = 0.049), GABA+ /tCr (*p* = 0.047), and tCho levels (*p* = 0.033) among WMHs subgroups and HCs group.

For the multiple comparisons, both mild (*p* = 0.047) and moderate-severe (*p* = 0.037) WMHs subgroups had significantly lower GABA+ levels compared to HCs group (Tables 2, 3 and Figure 2C). No significant difference in GABA+ levels between moderate-severe WMHs and mild WMHs subgroups was found (*p* = 0.537) (Table 4 and Figure 2C). Metabolite relative ratio showed that moderate-severe WMHs subgroup (*p* = 0.025) had significantly lower GABA+ /tCr compared to HCs group but mild WMHs subgroup did not (*p* = 0.062) (Supplementary Tables 3–5 and Supplementary Figure 1B).

Moderate-severe WMHs subgroup had significantly increased tCho concentration when compared to HCs group (*p* = 0.019) (Table 3 and Figure 2D). However, there was no significant difference in tCho concentration between mild WMHs subgroup and HCs group (*p* = 0.769) (Table 2 and Figure 2D).

## Discussion

In this study, the brain GABA+ levels of 24 WMHs patients and 20 healthy controls were assessed with *J*-edited MR spectroscopy. Further, the levels of other major metabolites (including Glx, tNAA, tCho, and tCr) have also been analyzed. This study demonstrates significant reductions of GABA+ levels in centrum semiovale in WMHs patients when compared to age and gender-matched healthy controls. In addition, we found that tCho levels were elevated in moderate-severe WMHs subgroup patients compared to healthy controls.

The spectroscopic volumes that were chosen for edited MR spectroscopy in this study was centrum semiovale. This is because centrum semiovale is the area of the so-called boundary zone positioned distal to the deep and superficial branches of the middle cerebral artery. This territory is particularly susceptible to inadequate blood perfusion or ischemia secondary to risk elements associated with the deep vascular region, and this region of interest is frequently chosen in patients with cerebral small vessel disease (Nitkunan et al., 2006, 2009).

The primary finding of this study is that the GABA+ level estimated from *J*-edited MRS using MEGA-PRESS was substantially lower in WHMs patients when compared to healthy controls. GABA is the primary inhibitory neurotransmitter in the central nervous system and plays an important role in effective information processing in the brain (Bartos et al., 2007) as well as normal behavioral performance or cognitive function (Boy et al., 2011). Therefore, reduced GABA+ concentrations identified in this study may be underlying pathological mechanisms for the concealed cognitive deficits in WMHs patients, as reported in individuals with cerebral small vessel disease (Zanon Zotin et al., 2021). In the subgroup analysis, lower levels of GABA+ were also detected both in moderate-severe and mild WMHs subgroup patients compared to HCs. This may imply that abnormalities in GABA+ levels develop early in the disease, and variations in GABA+ levels have a high sensitivity for studying pathological processes in WMHs. Moreover, the gray matter tissue fraction showed a significant difference between moderate-severe WMHs subgroup and HCs but not between mild WMHs subgroup and HCs, which may suggest that metabolic changes precede structural brain changes. Further exploration of the reason for this phenomenon is needed.

Our findings also revealed that the level of tCho in the centrum semiovale was considerably higher in patients with moderate-severe WMHs than healthy controls. tCho is involved in the composition of cell membranes, reflecting cell metabolism and myelin formation. Increased tCho levels may reflect membrane breakdown or demyelination (De Stefano et al., 2005; Lin et al., 2005), and the elevated tCho levels in our study represent ischemic brain injury in patients with moderate-severe WMHs. Similarly, a previous study found patients with transient ischemic attack within 3 days of onset show an increase in choline (Bisschops et al., 2002). Ischemia or hypometabolism are also potential pathogenic mechanisms for LA (Sun et al., 2022). In the subgroup analysis, the level of tCho showed no significant increase in mild WMHs subgroup patients compared to healthy controls, which suggests that mild cerebral white matter hyperintensities do not cause changes in the level of tCho until the disease has progressed to a certain level.

Interestingly, we did not find a significant difference in the level of tNAA between patients with WMHs patients and healthy controls, which contract to the results of the previous study (Wang et al., 2012), which may be a result of the small sample size, the severity of white matter lesions, or the clinical heterogeneity. In addition, given the varying severity of WMHs, part of moderate-severe patients with frontal-parietal as well as paraventricular demyelination contains white matter lesion within the MRS voxels, and the inclusion of demyelinating lesions within the voxels may affect metabolite quantification outcomes, which needs further investigation. It is equally worthy of attention that a significant difference in hypertension was found between the WMHs patients and healthy controls, this may indicate that WMH patients are more likely to have concomitant hypertension than healthy individuals, in other words, hypertension is one of the underlying pathogenic mechanisms in WMHs (Markus and de Leeuw, 2023). Furthermore, it has been shown that intensive blood pressure lowering slows the progression of WHMs (Nasrallah et al., 2019). Our findings to some extent reveal potential metabolic markers of WMHs; in future studies, we will further explore which high-risk factors are associated with metabolic changes in WMHs patients to better prevent the occurrence of WHMs in individuals.

## Limitation

Our study has some limitations that need to be discussed. Firstly, previous studies have shown that GABA concentrations vary with location (Evans et al., 2010; Gao et al., 2013), thus further studies should be performed to explore whether there are also differences in GABA concentration in other different brain regions (e.g., the right centrum semiovale, the frontal lobe, the temporal lobe or hippocampus). Secondly, the edited GABA signal contains a significant contribution from co-edited macromolecules and homocarnosine. A previous study has developed a new method for macromolecule suppression to extract pure GABA (Edden et al., 2012), but the technique has practical limitations: MM-only spectra is time-consuming and the signal-to-noise ratio of GABA may be degraded; acquiring MM-suppressed edited experiments makes the acquisition substantially more sensitive to field changes. Additionally, for the majority of applications, variations in macromolecules between subjects are slightly compared with the variation in GABA levels (Kreis et al., 2005). Homocarnosine concentration is much less than GABA, and its impact on the results can be ignored. Comprehensively considered, GABA+ measurement is more appropriate. Finally, our results show the moderate-severe WMH group has a decreased mean GABA+ levels but increased median GABA+ levels than mild WMH group, which may be due to our modest sample size. Thus, future studies with a larger cohort of participants will boost the stability of our study results.

## Conclusion

In conclusion, we found reduced GABA+ levels in WMHs patients and increased tCho levels in moderate-severe WMHs subgroup patients compared to healthy controls. These findings support the potential for MRS to establish GABA+ and tCho measurements as clinically relevant metabolic markers for WMHs, although further research efforts are needed to better understand the findings reported here.

## Data availability statement

The raw data supporting the conclusions of this article will be made available by the authors, without undue reservation.

## Ethics statement

The studies involving humans were approved by The First Affiliated Hospital of Zhengzhou University. The studies were conducted in accordance with the local legislation and institutional requirements. The participants provided their written informed consent to participate in this study.

## Author contributions

XW: Writing – original draft, Writing – review and editing. CW: Supervision, Writing – original draft, Writing – review and editing. PM: Resources, Writing – original draft. YW: Resources, Writing – original draft. LL: Conceptualization, Software, Writing – review and editing. ZL: Supervision, Writing – review and editing. YZ: Supervision, Validation, Writing – review and editing. JC: Funding acquisition, Writing – review and editing. CR: Supervision, Writing – review and editing.
