# Supplementary material for: Reduced GABA concentration in patients with white matter hyperintensities
Source: Front Neurosci. 2023 Dec 14;17:1320247. doi: 10.3389/fnins.2023.1320247 (PMC10752961; doi:10.3389/fnins.2023.1320247)
Supplement: Supplementary file 1 [file Data_Sheet_1.docx]

***Supplementary Material***

**Reduced GABA Concentration in Patients with White Matter Hyperintensities**

Xin Wang^1†^, Caihong Wang^1^^†*^, Peifang Miao^1^, Ying Wei^1^, Liangjie Lin^3^, Zhen Li^2^, Yong Zhang^1^, Jingliang Cheng^1^, Cuiping Ren^1*^

^*^**Correspondence:** Caihong Wang, No.1 Jianshe Dong Road, Erqi district, Zhengzhou 450052, China. Emails: [fccwangch@zzu.edu.cn;](mailto:fccwangch@zzu.edu.cn:) Cuiping Ren, No.1 Jianshe Dong Road, Erqi district, Zhengzhou 450052, China. Emails: [rcp810@sohu.com.](mailto:fccwangch@zzu.edu.cn:)

**Table1.** Hypertension, hyperlipidemia, diabetes mellitus, and cognitive dysfunction information for WMHs patients and healthy controls

|  | WMHs | HCs | P-value |
| --- | --- | --- | --- |
| number | 24 | 20 | —— |
| hypertension (%) | 6(25) | 0(0) | 0.025 |
| hyperlipidemia (%) | 3(13) | 0(0) | 0.239 |
| diabetes mellitus (%) | 2(8) | 0(0) | 0.493 |
| cognitive dysfunction (%) | 0(0) | 0(0) | —— |

HCs = healthy controls, WMHs = white matter hyperintensities

**Table2.** Metabolite relative ratio in WMHs patients and healthy controls

|  | WMHs | HCs | Test Statistics | P-value |
| --- | --- | --- | --- | --- |
| GABA+/tCr | 0.09±0.01 | 0.10±0.01 | t=-2.528 | 0.015 |
| GABA+/tNAA | 0.23±0.02 | 0.24±0.03 | t=-1.678 | 0.101 |
| Glx/tCr | 0.10±0.01 | 0.09±0.01 | t=1.181 | 0.244 |
| Glx/tNAA | 0.81±0.22 | 0.77±0.21 | t=0.560 | 0.579 |

GABA+ = GABA plus co-edited macromolecules, HCs = healthy controls, WMHs = white matter hyperintensities

**Table3.** Metabolite relative ratio in mild WMHs subgroup patients and healthy controls

|  | mild WMHs | HCs | Test Statistics | P-value |
| --- | --- | --- | --- | --- |
| GABA+/tCr | 0.09±0.01 | 0.10±0.01 | t=-1.936 | 0.062 |
| GABA+/tNAA | 0.23±0.03 | 0.24±0.03 | t=-1.136 | 0.265 |
| Glx/tCr | 0.10±0.01 | 0.09±0.01 | t=1.032 | 0.31 |
| Glx/tNAA | 0.79±0.26 | 0.77±0.21 | t=0.169 | 0.867 |

GABA+ = GABA plus co-edited macromolecules, HCs = healthy controls, WMHs = white matter hyperintensities

**Table4.** Metabolite relative ratio in moderate-severe WMHs subgroup patients and healthy controls

|  | moderate-severe WMHs | HCs | Test Statistics | P-value |
| --- | --- | --- | --- | --- |
| GABA+/tCr | 0.09±0.01 | 0.10±0.01 | t=-2.364 | 0.025 |
| GABA+/tNAA | 0.22±0.02 | 0.24±0.03 | t=-1.613 | 0.119 |
| Glx/tCr | 0.10±0.01 | 0.09±0.01 | t=1.113 | 0.275 |
| Glx/tNAA | 0.84±0.13 | 0.77±0.21 | t=0.945 | 0.353 |

GABA+ = GABA plus co-edited macromolecules, HCs = healthy controls, WMHs = white matter hyperintensities

**Table5.** Metabolite relative ratio in mild and moderate-severe WMHs subgroup patients

|  | mild WMHs | moderate-severe WMHs | Test Statistics | P-value |
| --- | --- | --- | --- | --- |
| GABA+/tCr | 0.09±0.01 | 0.09±0.01 | t=-0.541 | 0.595 |
| GABA+/tNAA | 0.23±0.03 | 0.22±0.02 | t=-0.535 | 0.598 |
| Glx/tCr | 0.10±0.01 | 0.10±0.01 | t=0.223 | 0.826 |
| Glx/tNAA | 0.79±0.26 | 0.84±0.13 | t=0.598 | 0.556 |

GABA+ = GABA plus co-edited macromolecules, HCs = healthy controls, WMHs = white matter hyperintensities


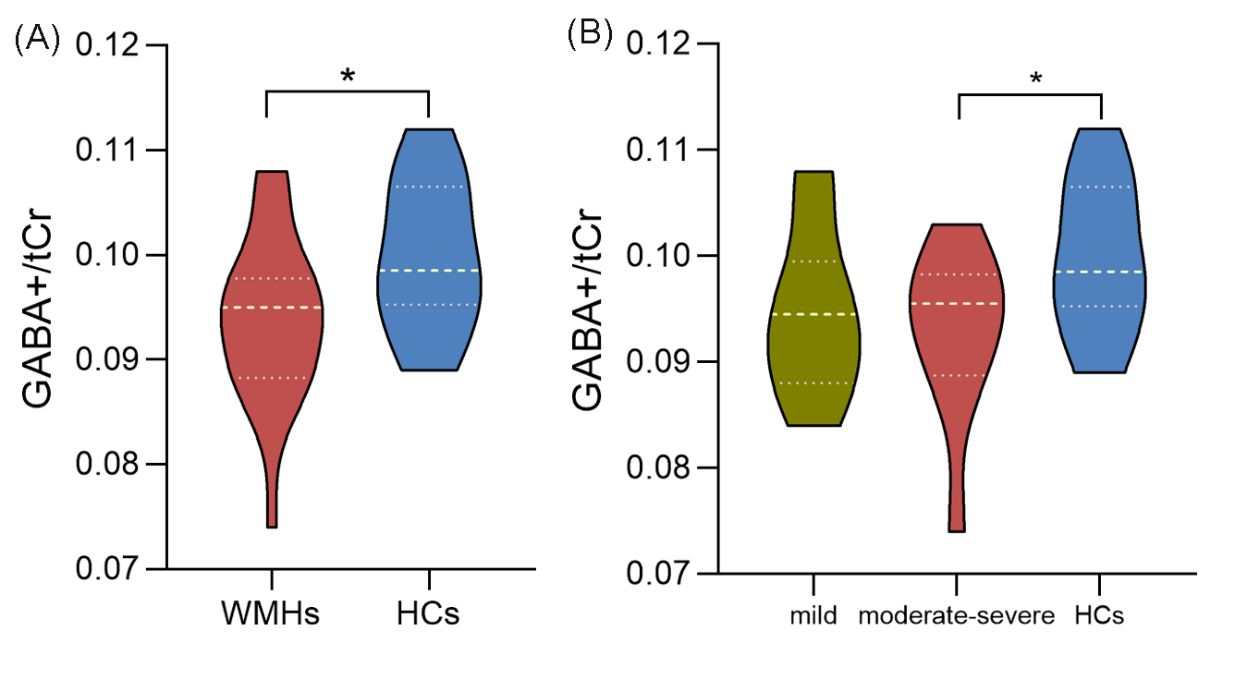


**Figure 1** GABA+/tCr of the WMHs group are significantly decreased compared to HCs (A). For comparison of GABA+/tCr levels within WMHs subgroups and HCs, moderate-severe WMHs subgroup had significantly decreased GABA+/tCr compared to HCs group (B). ^*^P<0.05.
